# Sleep Characteristics and Cancer-Related Outcomes: An Umbrella Review of Systematic Reviews and Meta-Analyses of Observational Studies

**DOI:** 10.3390/jcm11247289

**Published:** 2022-12-08

**Authors:** Xiaoying Li, Donghui Huang, Fanghua Liu, Xinyu Li, Jiale Lv, Qijun Wu, Yuhong Zhao

**Affiliations:** 1 Department of Clinical Epidemiology, Shengjing Hospital of China Medical University, Shenyang 110004, China; 2 Clinical Research Center, Shengjing Hospital of China Medical University, Shenyang 110004, China; 3 Liaoning Key Laboratory of Precision Medical Research on Major Chronic Disease, Shenyang 110004, China; 4 Department of Obstetrics and Gynecology, Shengjing Hospital of China Medical University, Shenyang 110004, China; 5 Key Laboratory of Reproductive and Genetic Medicine, China Medical University, National Health Commission, Shenyang 110004, China

**Keywords:** cancer, sleep, systematic review and meta-analysis, umbrella review

## Abstract

Background: Sleep is closely related to various diseases. Several meta-analyses have provided evidence of sleep and cancer, and yet the credibility of this evidence has not been comprehensively quantified. Thus, we conducted an umbrella review to quantify the evidence for systematic reviews and meta-analyses of observational studies on sleep characteristics (sleep duration, sleep quality, napping, bedtime, and wake-up time) and cancer-related outcomes. Methods: PubMed, Web of Science (Core Collection), and Embase databases were searched from inception until 29 July 2022. Assessment of Multiple Systematic Reviews, version 1, was used to evaluate the methodological quality of each eligible systematic review or meta-analysis. For each association, the summary effect with a 95% confidence interval was evaluated by fixed and random effects models. The 95% prediction interval, heterogeneity, small-study effects, and excess significance bias were also evaluated. Evidence of the associations from systematic reviews and meta-analyses was ranked based on the established criteria of published literature as convincing, highly suggestive, suggestive, weak, or non-significant. Results: The umbrella review identified thirty meta-analyses on the aforementioned associations from six articles. The methodological quality of five articles was high or moderate. Suggestive evidence was found for associations between long sleep duration and a 21% increased risk of colorectal cancer, a 9% increased all-cancer mortality and a 65% increased mortality of lung cancer, and associations between short sleep duration and a 21% increased mortality of lung cancer. Additionally, the evidence of associations between short sleep duration and lung cancer mortality was upgraded to convincing, and between long sleep duration and lung cancer mortality was upgraded to highly suggestive, among the population reporting 24 h sleep duration. Conclusion: Abnormal sleep duration might be linked to several adverse cancer-related outcomes.

## 1. Introduction

Cancer has always been a major public health hazard worldwide [1,2]. Approximately 19.3 million new cancer cases and 10.0 million cancer deaths occurred in 2020 across the globe [2]. The social burden of cancer is expected to continue to rise due to certain reasons, such as aging and changes in the distribution of modifiable risk factors [3]. Sleep characteristics, such as sleep duration, sleep quality, and napping, are emerging as potentially modifiable cancer risk factors of note [4].

The exact mechanisms underlying the association between sleep characteristics and cancer are unclear, but several potential mechanisms have been postulated. First, abnormal sleep could lead to a disturbance of circadian rhythm, which is linked to a variety of diseases, including cancers [5,6]. The disruption of circadian rhythm is related to deregulated cell proliferation, and previous experiments in vivo have found that cancer can develop in animal models of circadian disruption [7]. Meanwhile, population observational studies have found that shift work may be associated with the occurrence and progression of a variety of tumors, such as breast cancer [8], ovarian cancer [9], and lung cancer [10]. Second, abnormal sleep might cause a decrease in the body’s level of melatonin. Melatonin may interfere with various cancer hallmarks, such as evading growth suppressors, sustained proliferation, replication immortality, metastasis, resisting cell death, and angiogenesis, to suppress the onset and progression of cancer [11,12]. Third, long sleep duration might be associated with elevated inflammation level [13,14], which plays a vital role in the etiology and progression of cancer [15,16,17]. Meanwhile, short sleep duration could be associated with being overweight or obese [18,19], which might increase cancer risk through several means, such as causing inflammation and insulin resistance [20,21,22]. Nevertheless, sleep might also be a proxy of many physiological, social, and environmental factors that could affect morbidity and mortality from a variety of diseases [23].

An increasing number of observational studies on sleep characteristics and cancer have been published, and several systematic reviews and meta-analyses have summarized the evidence for this topic [24,25,26]. However, to our best knowledge, there has been little attempt to quantify the quality of this evidence. The umbrella review gives a structured quantitative method of the evidence from multiple systematic reviews and meta-analyses on the same topic and can grade the evidence based on specific criteria, such as the strength and precision of associations and assessment of biases [27]. An umbrella review by Gao et al. graded the evidence from meta-analyses of prospective studies on sleep and health outcomes [28]. However, certain sleep characteristics, such as napping, were not considered, and some cancers, such as colorectal cancer, lung cancer, ovarian cancer, and endometrial cancer, were not included in their umbrella review. That is partly because they focused on all health outcomes and, therefore, did not extract and evaluate the data of subgroup analyses by cancer type in systematic reviews or meta-analyses [28].

Thus, we conducted this umbrella review to systematically and comprehensively identify systematic reviews and meta-analyses of observational studies on sleep characteristics (sleep duration, sleep quality, napping, bedtime, and wake-up time) and cancer-related outcomes, summarize their findings, evaluate, and grade the quality of their evidence.

## 2. Methods

Reporting of this umbrella review adhered to the Preferred Reporting Items for Systematic Reviews and Meta-Analyses (PRISMA) reporting guidelines and the Meta-analysis of Observational Studies in Epidemiology (MOOSE) reporting guidelines [29,30]. The protocol of this study was registered at the International Prospective Register of Systematic Reviews (registration number CRD42022360686).

### 2.1. Literature Search

We conducted a systematic literature search in PubMed, Web of Science (Core Collection), and Embase databases from inception until 29 July 2022. The search strategy combined terms related to sleep, cancer, systematic review, and meta-analysis. The thesaurus terms were searched by the MeSH database of the PubMed database and were supplemented by reviewing the search strategy of relevant systematic reviews and meta-analyses. The detailed search strategy was shown in Table S1. No language restrictions were applied during the literature search. Additionally, we manually checked the reference of qualified systematic reviews and meta-analyses to avoid omission.

### 2.2. Eligibility Criteria

Eligible articles met the following criteria: (1) systematic reviews or meta-analyses on sleep characteristics (including sleep duration, sleep quality, napping, bedtime, and wake-up time) and cancer; (2) the subjects were human; (3) primary studies in systematic reviews or meta-analyses were observational studies; (4) the articles provided effect sizes with 95% confidence intervals (CIs) of per primary studies, such as relative risk (RR), odds ratio (OR), and hazard ratio (HR); and (5) the articles provided the number of cases or outcomes and the number of controls or all participants of per primary studies. Definitions of vocabularies covered in inclusion criteria were listed in Table S2.

Articles were excluded when meeting the following criteria: (1) the exposure factors were not sleep characteristics or the outcomes were not cancer-related events; (2) the research factors were not sleep characteristics that we were interested in, such as insomnia, sleep disturbance, and obstructive sleep apnea; (3) the articles were without quantitative synthesis; (4) the articles included family-based primary studies; (5) included primary datasets were less than 3; and (6) the articles had abstracts only, letters to editors, editorial comments, or unpublished articles.

When eligible systematic reviews or meta-analyses for the same research topic were over one, we selected the one with the largest dataset. If one eligible systematic review or meta-analysis included several research topics, we evaluated each topic separately. Two authors (X.L. (Xiaoying Li) and D.H.) independently screened eligible articles, and a third author (Q.W.) was responsible for quality control and the resolution of discrepancies.

### 2.3. Data Extraction

Two authors (X.L. (Xiaoying Li) and D.H.) independently extracted data from eligible articles, and a third author (Q.W.) was responsible for quality control and the resolution of discrepancies. The following data were extracted: (1) the first author; (2) the publication year; (3) the sleep characteristics and cancer-related outcomes; (4) the comparative method of sleep characteristics, such as long sleep duration compared with moderate sleep duration; (5) the number of included primary datasets; (6) the maximally adjusted effect sizes with 95% CIs, epidemiological design, detection methods of sleep characteristics, the Newcastle–Ottawa Scale (NOS) score, and the region of the primary study; and (7) the number of cases or outcomes and the number of controls or all participants in the primary study.

### 2.4. Methodological Quality Appraisal

Two authors (X.L. (Xiaoying Li) and D.H.) independently applied the Assessment of Multiple Systematic Reviews, version 1 (AMSTAR-1) tool to evaluate the methodological quality of each eligible systematic review or meta-analysis. And a third author (Q.W.) was responsible for quality control and the resolution of discrepancies. The AMSTAR-1 tool involves 11 items that can be scored 0 or 1, for a total of 11 points. Values of 8–11, 4–7, and 0–3 points are rated as high, medium, and low quality, respectively [31].

### 2.5. Data Analysis

We repeated all meta-analyses of the included review with the largest sample. For each eligible systematic review or meta-analysis, the summary effects with 95% CIs were re-calculated through both fixed-effects and random-effects models [32,33]. *I*^2^ metrics were used to quantify the heterogeneity between studies [34], which was considered large when *I*^2^ exceeded 50%. Meanwhile, we calculated the 95% CI of *I*^2^ and assessed the heterogeneity between studies by using the Cochran Q statistic with *p* < 0.10 as statistically significant [33]. We further calculated a 95% prediction interval (PI) of the summary of random effects, which can explain the degree of heterogeneity between studies and indicate the uncertainty of the effect if future studies focus on the same topic [35,36].

We calculated the standard error (SE) of the effect size for each primary study to identify the study with the smallest SE in the eligible systematic review or meta-analysis. Egger’s regression asymmetry test was used to assess small-study effects (i.e., smaller studies tended to give larger effect sizes in comparison with larger studies) [37]. Small-study effects were judged to exist when the following two aspects were simultaneously satisfied: (1) *p*_Egger_ < 0.10 and (2) the effect of the largest study (i.e., the smallest SE) was more conservative than in the random-effects meta-analysis [38]. Further, we conducted an excess significance test to assess whether the observed number of studies (O) with significant results (positive studies, *p* < 0.05) differed from the expected number (E) in a meta-analysis [39]. A combination of *p* < 0.10 and O > E indicated excess significance bias.

We further conducted subgroup analyses for each eligible systematic review or meta-analysis based on region (North America, Asia, and Europe) and sleep duration type (sleep duration at night and sleep duration during 24 h; only if the exposure factor was sleep duration). All analyses were performed by Stata 11 software (Stata LLC, College Station, TX, USA), with a two-sided test and *p* < 0.05 as statistically significant (except for special instructions).

### 2.6. Quality Evaluation of Evidence

Based on previously published umbrella reviews, we graded the quality of the evidence for each eligible systematic review and meta-analysis, as well as its subgroup analyses [40,41,42]. In brief, the quality of evidence was graded as convincing, highly suggestive, suggestive, and weak, if the summary random effects were statistically significant (detailed in Table 1).

### 2.7. Sensitivity Analysis

We performed sensitivity analysis through two methods. First, for systematic reviews or meta-analyses that included both cohort and case-control studies, re-analysis and re-evaluation were conducted after case-control studies were removed. Then, if eligible systematic reviews or meta-analyses for the same research topic were over one, we selected the one with the second largest dataset to re-analyze and re-evaluate.

## 3. Results

### 3.1. Selection and Quality Appraisal of Articles

**Figure 1:**
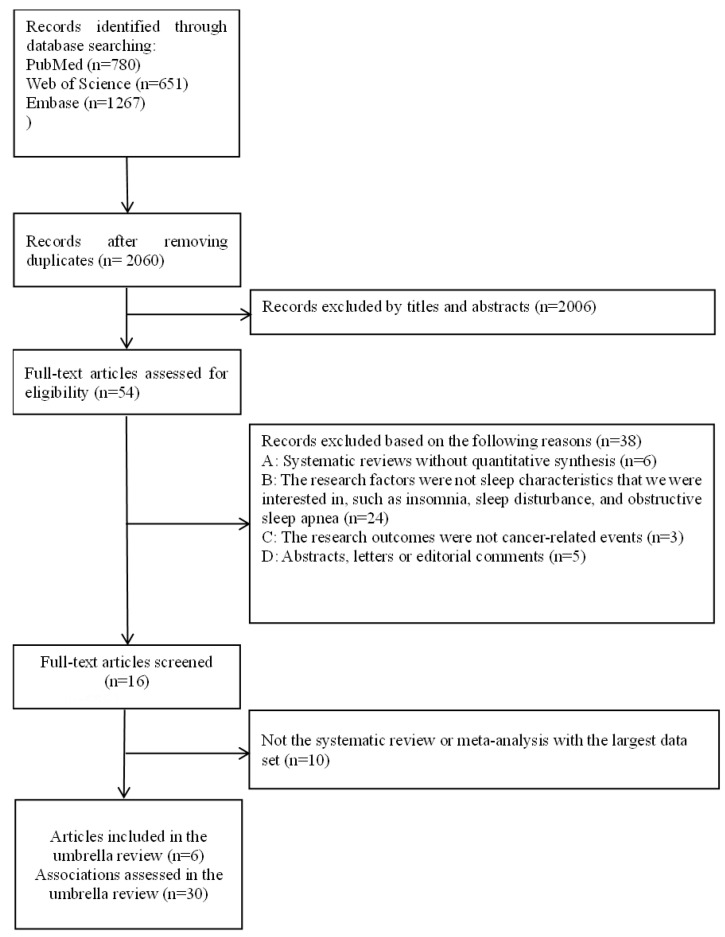
Flowchart of the literature search and screening.

**Figure 2:**
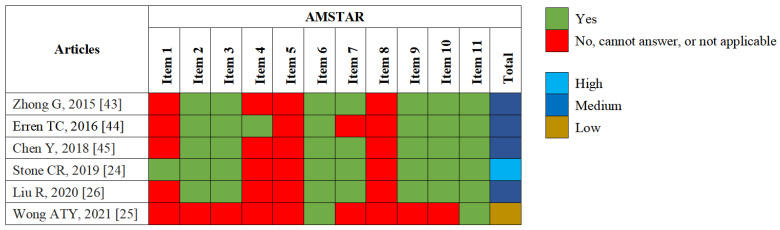
Methodological quality appraisal of each eligible systematic review or meta-analysis with AMSTAR-1. Items: 1. Was an “a priori” design provided? 2. Was there duplicate study selection and data extraction? 3. Was a comprehensive literature search performed? 4. Was the status of publication (i.e., grey literature) used as an inclusion criterion? 5. Was a list of studies (included and excluded) provided? 6. Were the characteristics of the included studies provided? 7. Was the scientific quality of the included studies assessed and documented? 8. Was the scientific quality of the included studies used appropriately in formulating conclusions? 9. Were the methods used to combine the findings of studies appropriate? 10. Was the likelihood of publication bias assessed? 11. Was the conflict of interest stated?

In total, we identified 2698 records, scrutinized 54 full-text articles, and excluded 48 full-text articles, which were listed in Supplementary File S1. Ultimately, six articles [24,25,26,43,44,45] were included in this umbrella review, which referred to 30 meta-analyses of associations between sleep characteristics and cancer-related outcomes (Figure 1). These six articles were published between 2015 and 2021, of which five (83.33%) were rated as medium or high quality and one (16.67%) was rated as low quality, based on the AMSTAR-1 tool (Figure 2). The shortcomings of the methodological quality of these articles mainly included: no “priori” design was provided, grey literature was not considered in literature retrieval, no list of excluded studies was provided, and the scientific quality of the included studies was not appropriately applied to formulate conclusions (Figure 2). Of the six articles, four evaluated the quality of primary articles using the NOS score, and all their primary articles were of medium or high quality. Nevertheless, the other two articles did not evaluate the quality of the primary articles.

### 3.2. Basic Characteristics of Meta-Analyses

Table S3 summarized the basic characteristics of 30 meta-analyses on sleep characteristics and cancer-related outcomes, which included primary cohort and case-control studies. Only sleep duration, sleep quality, and napping were included as exposure factors because no eligible systematic review or meta-analysis of bedtime/wake-up time and cancer-related outcomes was found. Detection methods of sleep characteristics were mainly questionnaires or interviews, and very few primary studies used sleep watch actigraphy. In primary studies, short and long sleep duration was defined in a variety of ways. Short sleep duration was defined as 3–5, 3–6, <5, ≤5, <5.9, <6, ≤6, ≤6.5, <7, and ≤7 h per night/24 h. Long sleep duration was defined as >7, >8, ≥8, >9, ≥9, >10, ≥10, >10.2, and 10–12 h per night/24 h. As a reference, moderate sleep duration was defined as 5–8, 6–7, 6–8, 6.1–8.9, 6.6–7.4, 7, 7–7.5, 7–7.9, 7–8, 7–9, 8, and 8–9 h per night/24 h. A wide range of cancer-related outcomes was researched, including cancer risk (breast, colorectal, skin, lung, prostate, ovarian, endometrial, thyroid, and all cancer), as well as cancer mortality (breast, colorectal, lung, prostate, and all cancer). The median number of datasets and participants of the 30 meta-analyses was 6 (range: 3–65) and 428,243 (range: 92,059–6,609,205), respectively. The number of cases/outcomes was over 1000 in 26 meta-analyses (Table 2).

### 3.3. Findings

Summaries of 30 examined associations were provided in Table 2 and Table S3. There were nine associations (30.00%) with large heterogeneity (*I*^2^ > 50%) and only two associations (6.67%) with a 95% PI excluding the null value. Three associations (10.00%) were found to have small study effects and no excess significance bias was identified. We found suggestive evidence that long sleep duration was associated with a 21% higher colorectal cancer risk (95% CI: 1.08–1.34), a 9% higher all-cancer mortality (95% CI: 1.04–1.13), and a 65% higher lung cancer mortality (95% CI: 1.36–2.00), compared with moderate sleep duration. Meanwhile, the association between short sleep duration and a 21% increased lung cancer mortality was supported by suggestive evidence (95% CI: 1.10–1.33), compared with moderate sleep duration. Additionally, we found weak evidence that short sleep duration was linked to a decreased skin cancer risk compared with moderate sleep duration, and poor sleep quality was linked to an increased all-cancer risk compared with good sleep quality. However, we failed to find significant associations between short sleep duration and colorectal cancer risk, all-cancer mortality, between long sleep duration and skin cancer risk, and between short/long sleep duration and all-cancer risk, ovarian cancer risk, endometrial cancer risk, thyroid cancer risk, lung cancer risk, prostate cancer risk/mortality, and breast cancer risk/mortality, compared with moderate sleep duration. We also failed to find significant associations between sleep quality and breast cancer risk, and between napping and all-cancer risk/mortality. We performed sensitivity analyses and found all associations retained the same evidence ranking [44,45,46,47] (Table S4).

Most results of subgroup analyses were consistent with the main findings (Table 3 and Figure S1). Remarkably, in the 24 h sleep duration subgroup, the evidence of associations between short sleep duration and lung cancer mortality was upgraded from suggestive to convincing, and between long sleep duration and lung cancer mortality was upgraded from suggestive to highly suggestive. Additionally, associations of short sleep duration with all-cancer risk in the Asian subgroup and all-cancer mortality in the North American group were upgraded from non-significant to weak evidence. However, several associations between sleep characteristics and cancer-related outcomes were downgraded from suggestive or weak evidence to weak or non-significant evidence in certain subgroups.

## 4. Discussion

This is an umbrella review that quantitatively evaluates the existing evidence of associations between sleep characteristics and cancer-related outcomes based on systematic reviews and meta-analyses of observational studies. Overall, thirty associations were evaluated, six of which were statistically significant, four of which were supported by suggestive evidence, and two of which were supported by weak evidence. Suggestive evidence was found for associations between long sleep duration and colorectal cancer risk, all-cancer mortality, lung cancer mortality, and the association between short sleep duration and lung cancer mortality. Interestingly, subgroup analyses found that the evidence of associations between short sleep duration and lung cancer mortality was upgraded from suggestive to convincing, and that between long sleep duration and lung cancer mortality was upgraded from suggestive to highly suggestive in the 24 h sleep duration subgroup.

A previous umbrella review by Gao et al. evaluated the evidence of sleep and health outcomes based on meta-analyses of prospective observational studies [28]. For sleep and cancer, however, only associations between sleep duration and all-cancer risk, breast cancer risk, all-cancer mortality, and prostate cancer mortality were assessed in their umbrella review. The relevant findings were consistent with the current umbrella review [28]. By contrast, this current umbrella review focused on a wider range of sleep characteristics than just sleep duration, included more cancer-related outcomes, and conducted subgroup analyses based on region and sleep duration type. In addition, in order to more comprehensively assess sleep characteristics and cancer-related outcomes, this present umbrella review was conducted based on systematic reviews and meta-analyses of all observational studies, not just prospective studies. Meanwhile, we performed sensitivity analyses by retaining only prospective studies and found consistent results.

We found the association between long sleep duration and increased mortality of lung cancer was highly significant in the random-effects models (*p* < 1 × 10^−6^). Nevertheless, the 95% PI of the summary random effect included the null value, indicating that the association might not exist in specific settings. *I*^2^ exceeded 50%, suggesting that large heterogeneity between studies existed. Moreover, *p* exceeded 0.05 in the largest study (i.e., the smallest SE) of the meta-analysis, so the evidence of associations between long sleep duration and increased mortality of lung cancer was only rated as suggestive. This suggestive evidence was upgraded to highly suggestive in the 24 h sleep duration subgroup, but was downgraded to non-significant in the night sleep duration subgroup. Meanwhile, suggestive evidence for the association between short sleep duration and increased lung cancer mortality was observed. That was upgraded to convincing in the 24 h sleep duration subgroup, but was downgraded to non-significant in the night sleep duration subgroup. However, there were only three datasets in the night sleep duration subgroup, so the results of the meta-analysis might need to be further validated. Therefore, well-designed studies are necessary to further investigate the association between night sleep duration and lung cancer mortality. Additionally, our findings suggested that associations of cancer-related outcomes with sleep duration and quality might vary according to the region.

To our best knowledge, the current umbrella review is the first to conduct a critical and comprehensive appraisal of the existing systematic reviews and meta-analyses of observational studies on sleep characteristics and cancer-related outcomes. We conducted numerous subgroup analyses by region and sleep duration type and found convincing and highly suggestive evidence in certain subgroups. Moreover, the AMSTAR-1 tool was used to evaluate the methodological quality of all included systematic reviews or meta-analyses, and most of them (83.33%) had medium or high quality. Additionally, we performed several sensitivity analyses by removing case-control studies, as well as assessing the systematic review or meta-analysis with the second largest dataset, and found all associations retained the same evidence ranking.

Despite its strengths, this umbrella review had several limitations that should be discussed. First, only existing systematic reviews and meta-analyses of observational studies were included in this umbrella review. Thus, some individual studies, as well as systematic reviews and meta-analyses of randomized controlled trials, might be ignored. However, this limitation might not affect our findings because systematic reviews and meta-analyses with the largest dataset were included in our work, and systematic reviews or meta-analyses of randomized controlled trials on the current research topic have not been reported. Second, one included article in our umbrella review had low quality based on the AMSTAR-1 tool. And the AMSTAR tool has several limitations which may result in an overly optimistic evaluation of the quality of reviews [48]. Moreover, two of the included six articles failed to evaluate the quality of the primary articles. Nevertheless, that might not reduce the reliability of findings on account of consistent results of sensitivity analysis through assessing the systematic review or the meta-analysis with the second largest data. Third, this work depended on systematic reviews and meta-analyses of observational studies so that the validity of results depended on the quality of these included observational studies. However, observational studies had common limitations, such as recall and confounding bias, self-reported sleep information, and misclassification. Moreover, we could not avoid the limitations of meta-analyses. For example, sleep duration was diversely categorized in primary studies, but was uniformly categorized as long, moderate, and short sleep duration in meta-analyses. Therefore, potential biases might exist. Fourth, many meta-analyses included less than 10 primary studies in this umbrella review, which might reduce the power of excess significance tests and Egger’s tests [49]. Fifth, although we conducted subgroup analyses by region and sleep duration type, some potential confounding factors, such as age and gender, failed to be considered directly in this work because relevant data were unavailable. However, we extracted the maximally adjusted effect size per primary study instead of crude effect sizes for analysis, in order to minimize the impact of confounding bias. Sixth, limitations with the search strategy led to inadequate retrieval and evaluation. Although the search strategy combined as many topic-related terms as possible, certain synonyms were missing (e.g., “meta-analyses”). Moreover, for the three databases, the same thesaurus terms were used. Although the thesaurus terms were searched by the MeSH database of the PubMed database, and were supplemented through reviewing the search strategy of relevant systematic reviews and meta-analyses, the “explode” function of the Embase database for the thesaurus terms was ignored. Lastly, assessments for associations of cancers with sleep quality and napping were rare in this work because relevant published systematic reviews and meta-analyses were limited. Additionally, associations of cancers with several sleep characteristics (bedtime and wake-up time) failed to be assessed due to the absence of relevant systematic reviews and meta-analyses.

## 5. Conclusions

The findings of the current umbrella review reinforced the preexisting understanding of associations between sleep and cancer. In this umbrella review, associations between sleep duration and colorectal cancer risk, all-cancer mortality, and lung cancer mortality were supported by suggestive evidence. Additionally, associations of lung cancer mortality with short and long sleep duration were supported by convincing evidence and highly suggestive, respectively, among the population reporting 24 h sleep duration. A broader range of sleep characteristics, rather than just sleep duration, should be considered as exposure factors in future research.

## Figures and Tables

**Table 1:** Criteria for quality-of-evidence classification.

Category	Criteria
Convincing (class 1)	(1) *p* < 10^−6^ in the random-effects model (2) >1000 cases/outcomes (3) *p* < 0.05 in the largest study * (4) *I^2^* < 50% (5) A 95% prediction interval excluding the null value (6) No evidence of small-study effects or excess significance bias
Highly suggestive (class 2)	(1) *p* < 10^−6^ in the random-effects model (2) >1000 cases/outcomes (3) *p* < 0.05 in the largest study *
Suggestive (class 3)	(1) *p* < 10^−3^ in the random-effects model (2) >1000 cases/outcomes
Weak (class 4)	(1) *p* < 0.05 in the random-effects model
Non-significant	(1) *p* > 0.05 in the random-effects model

* The largest study: the study with the smallest standard error in a meta-analysis.

**Table 2:** Evidence evaluation of associations between sleep characteristics and cancer-related outcomes.

Outcome	Source	Comparison	Quality Evaluation of Evidence							
			*p*_Random-Effects_	No. of Cases/Outcomes	*p*_the Largest Study_ *	*I^2^* (95% CI)	95% Prediction Interval	Small-Study Effects	Excess Significance Bias	Evidence Class
**Sleep duration**										
All-cancer risk	Chen Y, 2018 [45]	short vs. ref	>0.05	>1000	>0.05	29.8 (4, 49)	0.86, 1.18	no	no	Non-significant
		long vs. ref	>0.05	>1000	>0.05	31.3 (6, 50)	0.82, 1.26	no	no	Non-significant
Skin cancer risk	Chen Y, 2018 [45]	short vs. ref	<0.05 but >10^−3^	>1000	<0.05	0.0 (0, 75)	0.85, 1.02	no	no	Weak
		long vs. ref	>0.05	>1000	>0.05	19.4 (0, 64)	0.64, 1.34	no	no	Non-significant
Colorectal cancer risk	Chen Y, 2018 [45]	short vs. ref	>0.05	>1000	>0.05	38.3 (0, 75)	0.75, 1.46	no	no	Non-significant
		long vs. ref	<10^−3^ but >10^−6^	>1000	>0.05	0.0 (0, 75)	1.03, 1.41	no	no	Suggestive
Ovarian cancer risk	Chen Y, 2018 [45]	short vs. ref	>0.05	<1000	>0.05	44.3 (0, 83)	0.02, 46.34	no	no	Non-significant
		long vs. ref	>0.05	<1000	>0.05	60.5 (0, 89)	0.00, 567.95	no	no	Non-significant
Endometrial cancer risk	Chen Y, 2018 [45]	short vs. ref	>0.05	>1000	>0.05	50.0 (0, 86)	0.15, 6.20	yes	no	Non-significant
		long vs. ref	>0.05	>1000	>0.05	0.0 (0, 90)	0.23, 4.90	no	no	Non-significant
Thyroid cancer risk	Chen Y, 2018 [45]	short vs. ref	>0.05	<1000	>0.05	65.0 (0, 90)	0.00, 522.15	no	no	Non-significant
		long vs. ref	>0.05	<1000	>0.05	0.0 (0, 90)	0.06, 14.58	no	no	Non-significant
Lung cancer risk	Chen Y, 2018 [45]	short vs. ref	>0.05	>1000	>0.05	46.1 (0, 80)	0.65, 1.65	no	no	Non-significant
		long vs. ref	>0.05	>1000	>0.05	41.6 (0, 78)	0.59, 1.73	no	no	Non-significant
Prostate cancer risk	Liu R, 2020 [26]	short vs. ref	>0.05	>1000	>0.05	0.0 (0, 75)	0.88, 1.10	no	no	Non-significant
		long vs. ref	>0.05	>1000	>0.05	56.2 (0, 82)	0.56, 1.40	no	no	Non-significant
Breast cancer risk	Wong ATY, 2021 [25]	short vs. ref	>0.05	>1000	>0.05	7.5 (0, 44)	0.96, 1.03	no	no	Non-significant
		long vs. ref	>0.05	>1000	>0.05	11.2 (0, 49)	0.93, 1.07	yes	no	Non-significant
All-cancer mortality	Stone CR, 2019 [24]	short vs. ref	>0.05	>1000	>0.05	0.8 (0, 45)	0.99, 1.07	no	no	Non-significant
		long vs. ref	<10^−3^ but >10^−6^	>1000	>0.05	5.4 (0, 37)	1.02, 1.16	no	no	Suggestive
Lung cancer mortality	Stone CR, 2019 [24]	short vs. ref	<10^−3^ but >10^−6^	>1000	<0.05	58.4 (28, 76)	0.87, 1.66	no	no	Suggestive
		long vs. ref	<10^−6^	>1000	>0.05	84.5 (76, 90)	0.76, 3.58	no	no	Suggestive
Breast cancer mortality	Stone CR, 2019 [24]	short vs. ref	>0.05	>1000	>0.05	57.1 (0, 84)	0.53, 2.20	no	no	Non-significant
		long vs. ref	>0.05	>1000	<0.05	63.8 (5, 86)	0.30, 4.05	no	no	Non-significant
Prostate cancer mortality	Stone CR, 2019 [24]	short vs. ref	>0.05	>1000	>0.05	0.0 (0, 85)	0.74, 1.41	no	no	Non-significant
		long vs. ref	>0.05	>1000	>0.05	43.4 (0, 79)	0.35, 2.47	no	no	Non-significant
**Sleep quality**										
All-cancer risk	Erren TC, 2016 [44]	poor vs. good	<0.05 but >10^−3^	>1000	>0.05	55.2 (12,77)	0.91, 1.37	yes	no	Weak
Breast cancer risk	Erren TC, 2016 [44]	poor vs. good	>0.05	>1000	>0.05	25.7 (0, 72)	0.77, 1.40	no	no	Non-significant
**Napping**										
All-cancer risk	Erren TC, 2016 [44]	“yes” vs. “no”	>0.05	>1000	>0.05	85.4 (68, 93)	0.82, 1.30	no	no	Non-significant
All-cancer mortality	Zhong G, 2015 [43]	“yes” vs. “no”	>0.05	>1000	>0.05	8.9 (0, 86)	0.85, 1.35	no	no	Non-significant

Abbreviation: CI, confidence interval. * The largest study: the study with the smallest standard error in the meta-analysis.

**Table 3:** Subgroup analysis for evidence evaluation on sleep characteristics and cancer-related outcomes.

Outcome	Source	Comparison	Subgroup	No. of Datasets	Quality Evaluation of Evidence							
					*p*_Random-Effects_	No. of Cases/Outcomes	*p*_the Largest Study_ *	*I^2^* (95% CI)	95% Prediction Interval	Small-Study Effects	Excess Significance Bias	Evidence Class
**Sleep duration**												
All-cancer risk	Chen Y, 2018 [45]	short vs. ref	North America	55	>0.05	>1000	>0.05	13.2 (0, 38)	0.89, 1.08	no	no	Non-significant
			Europe	4	>0.05	>1000	<0.05	66.7 (3, 89)	0.29, 4.84	no	no	Non-significant
			Asia	5	<0.05 but >10^−3^	>1000	>0.05	58.1 (0, 84)	0.57, 3.24	yes	no	Weak
			24 h sleep	27	>0.05	>1000	>0.05	39.2 (3, 62)	0.86, 1.19	no	no	Non-significant
			Night sleep	38	>0.05	>1000	>0.05	23.3 (0, 49)	0.83, 1.21	no	no	Non-significant
		long vs. ref	North America	55	>0.05	>1000	>0.05	7.4 (0, 33)	0.92, 1.11	no	no	Non-significant
			Europe	4	>0.05	>1000	>0.05	70.6 (16, 90)	0.18, 5.00	no	no	Non-significant
			Asia	5	>0.05	>1000	<0.05	82.0 (59, 92)	0.11, 4.87	no	no	Non-significant
			24 h sleep	27	>0.05	>1000	>0.05	50.0 (22, 68)	0.70, 1.39	no	no	Non-significant
			Nigh sleep	38	>0.05	>1000	>0.05	9.6 (0, 39)	0.91, 1.15	no	no	Non-significant
Skin cancer risk	Chen Y, 2018 [45]	short vs. ref	North America	6	<0.05 but >10^−3^	>1000	<0.05	0.0 (0, 75)	0.85, 1.02	no	no	Weak
			24 h sleep	6	<0.05 but >10^−3^	>1000	<0.05	0.0 (0, 75)	0.85, 1.02	no	no	Weak
		long vs. ref	North America	6	>0.05	>1000	>0.05	19.4 (0, 64)	0.64, 1.34	no	no	Non-significant
			24 h sleep	6	>0.05	>1000	>0.05	19.4 (0, 64)	0.64, 1.34	no	no	Non-significant
Colorectal cancer risk	Chen Y, 2018 [45]	short vs. ref	North America	6	>0.05	>1000	>0.05	38.3 (0, 75)	0.75, 1.46	no	no	Non-significant
			24 h sleep	3	>0.05	>1000	>0.05	0.0 (0, 90)	0.44, 2.35	no	no	Non-significant
			Night sleep	3	>0.05	>1000	>0.05	65.5 (0, 90)	0.06, 17.79	no	no	Non-significant
		long vs. ref	North America	6	<10^−3^ but >10^−6^	>1000	>0.05	0.0 (0, 75)	1.03, 1.41	no	no	Suggestive
			24 h sleep	3	<0.05 but >10^−3^	>1000	>0.05	0.0 (0, 90)	0.37, 4.09	no	no	Weak
			Night sleep	3	<0.05 but >10^−3^	>1000	>0.05	23.4 (0, 92)	0.31, 4.59	no	no	Weak
Endometrial cancer risk	Chen Y, 2018 [45]	short vs. ref	North America	3	>0.05	>1000	>0.05	50.0 (0, 86)	0.15, 6.20	yes	no	Non-significant
		long vs. ref	North America	3	>0.05	>1000	>0.05	0.0 (0, 90)	0.23, 4.90	no	no	Non-significant
Thyroid cancer risk	Chen Y, 2018 [45]	short vs. ref	North America	3	>0.05	<1000	>0.05	65.0 (0, 90)	0.00, 522.15	no	no	Non-significant
			Night sleep	3	>0.05	<1000	>0.05	65.0 (0, 90)	0.00, 522.15	no	no	Non-significant
		long vs. ref	North America	3	>0.05	<1000	>0.05	0.0 (0, 90)	0.06, 14.58	no	no	Non-significant
			Night sleep	3	>0.05	<1000	>0.05	0.0 (0, 90)	0.06, 14.58	no	no	Non-significant
Lung cancer risk	Chen Y, 2018 [45]	short vs. ref	North America	4	>0.05	>1000	>0.05	0.0 (0, 85)	0.79, 1.26	no	no	Non-significant
			Night sleep	3	>0.05	>1000	>0.05	66.5 (0, 90)	0.04, 25.51	yes	no	Non-significant
		long vs. ref	North America	4	>0.05	>1000	>0.05	0.0 (0, 85)	0.71, 1.25	no	no	Non-significant
			Night sleep	3	>0.05	>1000	>0.05	69.9 (0, 91)	0.04, 28.74	no	no	Non-significant
Prostate cancer risk	Liu R, 2020 [26]	short vs. ref	North America	3	>0.05	>1000	>0.05	0.0 (0, 90)	0.55, 1.70	no	no	Non-significant
			24 h sleep	4	>0.05	>1000	>0.05	17.8 (0, 87)	0.64, 1.54	no	no	Non-significant
		long vs. ref	North America	3	>0.05	>1000	>0.05	33.9 (0, 93)	0.22, 3.89	no	no	Non-significant
			24 h sleep	4	>0.05	>1000	>0.05	71.0 (17, 90)	0.18, 3.40	no	no	Non-significant
Breast cancer risk	Wong ATY, 2021 [25]	short vs. ref	North America	10	>0.05	>1000	>0.05	1.1 (0, 63)	0.95, 1.02	no	no	Non-significant
			Asia	3	>0.05	<1000	>0.05	54.8 (0, 87)	0.07, 16.70	no	no	Non-significant
			24 h sleep	11	>0.05	>1000	>0.05	1.1 (0, 61)	0.97, 1.03	no	no	Non-significant
			Night sleep	4	>0.05	>1000	>0.05	0.0 (0, 85)	0.90, 1.05	no	no	Non-significant
		long vs. ref	North America	10	>0.05	>1000	>0.05	0.0 (0, 62)	0.94, 1.07	no	no	Non-significant
			Asia	3	>0.05	<1000	>0.05	0.0 (0, 90)	0.20, 3.29	no	no	Non-significant
			24 h sleep	11	>0.05	>1000	>0.05	20.7 (0, 60)	0.89, 1.11	yes	no	Non-significant
			Night sleep	4	>0.05	>1000	>0.05	0.0 (0, 85)	0.82, 1.18	no	no	Non-significant
All-cancer mortality	Stone CR, 2019 [24]	short vs. ref	North America	7	<0.05 but >10^−3^	>1000	>0.05	0.0 (0, 71)	0.99, 1.11	no	no	Weak
			Europe	6	>0.05	>1000	>0.05	0.0 (0, 75)	0.90, 1.31	no	no	Non-significant
			Asia	11	>0.05	>1000	>0.05	39.8 (0, 70)	0.84, 1.24	no	no	Non-significant
			24 h sleep	10	>0.05	>1000	>0.05	30.2 (0, 67)	0.90, 1.25	no	no	Non-significant
			Night sleep	14	>0.05	>1000	>0.05	0.0 (0, 55)	0.97, 1.06	no	no	Non-significant
		long vs. ref	North America	8	>0.05	>1000	>0.05	6.5 (0, 70)	0.95, 1.14	no	no	Non-significant
			Europe	7	>0.05	>1000	>0.05	0.0 (0, 71)	0.94, 1.29	no	no	Non-significant
			Asia	11	<10^−3^ but >10^−6^	>1000	>0.05	7.7 (0, 63)	1.03, 1.25	no	no	Suggestive
			24 h sleep	11	<0.05 but >10^−3^	>1000	>0.05	2.3 (0, 61)	1.01, 1.17	no	no	Weak
			Nigh sleep	15	<0.05 but >10^−3^	>1000	>0.05	13.4 (0, 51)	0.97, 1.23	no	no	Weak
Lung cancer mortality	Stone CR, 2019 [24]	short vs. ref	Asia	15	<10^−3^ but >10^−6^	>1000	<0.05	61.1 (32, 78)	0.86, 1.71	no	no	Suggestive
			24 h sleep	13	<10^−6^	>1000	<0.05	33.7 (0, 66)	1.03, 1.58	no	no	Convincing
			Night sleep	3	>0.05	>1000	>0.05	70.8 (1, 91)	0.06, 16.38	no	no	Non-significant
		long vs. ref	Asia	15	<10^−6^	>1000	>0.05	84.6 (76, 90)	0.78, 3.73	no	no	Suggestive
			24 h sleep	13	<10^−6^	>1000	<0.05	81.8 (70, 89)	0.87, 3.96	no	no	Highly suggestive
			Night sleep	3	>0.05	>1000	>0.05	0.0 (0, 90)	0.41, 2.82	no	no	Non-significant
Breast cancer mortality	Stone CR, 2019 [24]	short vs. ref	North America	3	>0.05	>1000	>0.05	77.8 (28, 93)	0.02, 50.93	yes	no	Non-significant
			Night sleep	4	>0.05	>1000	>0.05	67.0 (4, 89)	0.36, 3.13	no	no	Non-significant
		long vs. ref	North America	3	>0.05	>1000	<0.05	76.4 (23, 93)	0.00, 322.20	yes	no	Non-significant
			Night sleep	4	>0.05	>1000	<0.05	72.4 (22, 90)	0.15, 7.40	no	no	Non-significant
Prostate cancer mortality	Stone CR, 2019 [24]	long vs. ref	24 h sleep	3	>0.05	<1000	>0.05	0.0 (0, 90)	0.07, 21.17	no	no	Non-significant
**Sleep quality**												
All-cancer risk	Erren TC, 2016 [44]	poor vs. good	North America	4	>0.05	>1000	>0.05	72.0 (20, 90)	0.54, 2.40	no	no	Non-significant
			Europe	4	<0.05 but >10^−3^	>1000	>0.05	18.2 (0, 87)	0.56, 3.43	no	no	Weak
**Napping**												
All-cancer risk	Erren TC, 2016 [44]	“yes” vs. “no”	Europe	3	>0.05	>1000	>0.05	91.4 (78, 97)	0.41, 2.66	no	no	Non-significant

Abbreviation: CI, confidence interval. * The largest study: the study with the smallest standard error in the meta-analysis.

## Data Availability

Data are available upon request from the corresponding author.

## Supplementary Materials

The following supporting information can be downloaded at https://www.mdpi.com/article/10.3390/jcm11247289/s1, Table S1: Search strategy. Table S2: Definitions of vocabularies covered in inclusion criteria. Supplementary File S1: Lists of full-text articles excluded from the umbrella review. Table S3: Basic characteristics of meta-analyses that assess sleep characteristics and cancer-related outcomes. Table S4: Sensitivity analysis for evidence evaluation on sleep characteristics and cancer-related outcomes. Figure S1: Change of evidence class of associations between sleep characteristics and cancer-related outcomes in subgroup analyses compared to main findings. * Null value due to < 3 datasets in subgroups.
