# Supplementary material for: Sleep Characteristics and Cancer-Related Outcomes: An Umbrella Review of Systematic Reviews and Meta-Analyses of Observational Studies
Source: J Clin Med. 2022 Dec 8;11(24):7289. doi: 10.3390/jcm11247289 (PMC9785111; doi:10.3390/jcm11247289)
Supplement: Supplementary file 1 [file jcm-11-07289-s001.zip › jcm-2041796-supplementary.pdf]

Table S1. Search strategy

Databases (No. of reference)	Search strategy
**A) PubMed (n=780)**	((("Sleep"[Mesh]) OR (Sleeping) OR (Sleeping Habits) OR (Sleep Habits) OR (Habit, Sleep) OR (Habits, Sleep) OR (Sleep Habit) OR (Sleeping Habit) OR (Habit, Sleeping) OR (Habits, Sleeping) OR (Napping) OR (Nap) OR (Daytime sleep) OR (Daytime sleep) OR (Siesta) OR (Daytime sleepiness) OR (Daytime somnolence) OR (Bedtime) OR (Bed time) OR (Bed times) OR (Bedtimes) OR (Time of going to bed) OR (Wake up time) OR (Morning awakening time) OR (Normal awakening time) OR (Time of awakening) OR (Time of waking up)) AND ((Malignant neoplasm) OR (Tumor) OR (Neoplasm) OR (Tumors) OR (Neoplasia) OR (Neoplasias) OR (Cancer) OR (Cancers) OR ("Neoplasms"[Mesh]) OR (Malignancy) OR (Malignancies) OR (Malignant neoplasms) OR (Neoplasm, malignant) OR (Neoplasms, malignant) OR (Benign neoplasms) OR (Benign neoplasm) OR (Neoplasms, benign) OR (Neoplasm, benign) OR (malignant neoplasia) OR (malignant neoplastic disease) OR (malignant tumor) OR (malignant tumour) OR (neoplasia, malignant) OR (tumor, malignant) OR (tumour, malignant))) AND ((Systematic Review) OR (Meta-Analysis))
**B) Web of Science (Core Collection) (n=651)**	TS=((Sleep) OR (Sleeping) OR (Sleeping Habits) OR (Sleep Habits) OR (Habit, Sleep) OR (Habits, Sleep) OR (Sleep Habit) OR (Sleeping Habit) OR (Habit, Sleeping) OR (Habits, Sleeping) OR (Napping) OR (Nap) OR (Daytime sleep) OR (Siesta) OR (Daytime sleepiness) OR (Daytime somnolence) OR (Bedtime) OR (Bed time) OR (Bed times) OR (Bedtimes) OR (Time of going to bed) OR (Wake up time) OR (Morning awakening time) OR (Normal awakening time) OR (Time of awakening) OR (Time of waking up)) AND TS=((Malignant neoplasm) OR (Tumor) OR (Neoplasm) OR (Tumors) OR (Neoplasia) OR (Neoplasias) OR (Cancer) OR (Cancers) OR (Neoplasms) OR (Malignancy) OR (Malignancies) OR (Malignant neoplasms) OR (Neoplasm, malignant) OR (Neoplasms, malignant) OR (Benign neoplasms) OR (Benign neoplasm) OR (Neoplasms, benign) OR (Neoplasm, benign) OR (malignant

	neoplasia) OR (malignant neoplastic disease) OR (malignant tumor) OR (malignant tumour) OR (neoplasia, malignant) OR (tumor, malignant) OR (tumour, malignant)) AND TS=((Systematic Review) OR (Meta-Analysis))
**C) Embase (n=1267)**	((‘Sleep’/exp) OR ‘Sleeping’ OR ‘Sleeping Habits’ OR ‘Sleep Habits’ OR ‘Habit, Sleep’ OR ‘Habits, Sleep’ OR ‘Sleep Habit’ OR ‘Sleeping Habit’ OR ‘Habit, Sleeping’ OR ‘Habits, Sleeping’ OR ‘Napping’ OR ‘Nap’ OR ‘Daytime sleep’ OR ‘Siesta’ OR ‘Daytime sleepiness’ OR ‘Daytime somnolence’ OR (‘Bedtime’/exp) OR ‘Bed time’ OR ‘Bed times’ OR ‘Bedtimes’ OR ‘Time of going to bed’ OR (‘Wake up time’/exp) OR ‘Morning awakening time’ OR ‘Normal awakening time’ OR ‘Time of awakening’ OR ‘Time of waking up’) AND ((‘Malignant neoplasm’/exp) OR ‘Tumor’ OR ‘Neoplasm’ OR ‘Tumors’ OR ‘Neoplasia’ OR ‘Neoplasias’ OR ‘Cancer’ OR ‘Cancers’ OR ‘Neoplasms’ OR ‘Malignancy’ OR ‘Malignancies’ OR ‘Malignant neoplasms’ OR ‘Neoplasm, malignant’ OR ‘Neoplasms, malignant’ OR ‘Benign neoplasms’ OR ‘Benign neoplasm’ OR ‘Neoplasms, benign’ OR ‘Neoplasm, benign’ OR ‘malignant neoplasia’ OR ‘malignant neoplastic disease’ OR ‘malignant tumor’ OR ‘malignant tumour’ OR ‘neoplasia, malignant’ OR ‘tumor, malignant’ OR ‘tumour, malignant’) AND (‘systematic review’ OR ‘meta-analysis’)

Table S2. Definitions of vocabularies covered in inclusion criteria

**Vocabulary**	**Definition**
**Systematic review**	Systematic review is a secondary study of results from primary researches. Systematic reviews differ from traditional expert reviews and commentaries in that systematic reviews use a systematic, reproducible, and transparent approach which minimizes biases. [1]
**Meta-analysis**	Meta-analysis is referred to the statistical methods used to combine the results of primary studies. [1]
**Observational study**	A class of methods used to describe the distribution of disease or health condition in the population and to explore the relationship between exposure and disease by objectively recording the condition of subjects under study, by means of observation or interview, without imposing any intervention on subjects. It mainly includes the cross-sectional study, case-control study and cohort study. [2]

Reference

1. Li, L.M.; Zhan S.Y. Epidemiology, 7th ed.; People's Medical Publishing House: Beijing, China, 2012; pp. 359-374.

2. Li, L.M.; Zhan S.Y. Epidemiology, 7th ed.; People's Medical Publishing House: Beijing, China, 2012; pp. 1-103.

Supplementary file S1. Lists of full-text articles excluded from the umbrella review

A) Systematic reviews without quantitative synthesis (n=6)

1 Sigurdardottir, L. G. *et al.* Circadian disruption, sleep loss, and prostate cancer risk: A systematic review of epidemiologic studies. *Cancer Epidemiology Biomarkers and Prevention* **21**, 1002-1011, doi:10.1158/1055-9965.EPI-12-0116 (2012).

2 Malina, C., Frigo, S. & Mathelin, C. Sleep and breast cancer: Is there a link? *Gynecologie Obstetrique et Fertilite* **41**, 105-109, doi:10.1016/j.gyobfe.2012.12.008 (2013).

3 Wendeu-Foyet, M. G. & Menegaux, F. Circadian Disruption and Prostate Cancer Risk: An Updated Review of Epidemiological Evidences. *Cancer epidemiology, biomarkers & prevention : a publication of the American Association for Cancer Research, cosponsored by the American Society of Preventive Oncology* **26**, 985-991, doi:10.1158/1055-9965.Epi-16-1030 (2017).

4 Grgic, J. *et al.* Health outcomes associated with reallocations of time between sleep, sedentary behaviour, and physical activity: a systematic scoping review of isotemporal substitution studies. *International Journal of Behavioral Nutrition and Physical Activity* **15**, doi:10.1186/s12966-018-0691-3 (2018).

5 Gao, D. Q. & Wang, J. L. Current status of research on risk factors of lung cancer. *Chinese Journal of Cancer Prevention and Treatment* **26**, 1657-1662, doi:10.16073/j.cnki.cjcpt.2019.21.15 (2019).

6 Beverly Hery, C. M., Hale, L. & Naughton, M. J. Contributions of the Women's Health Initiative to understanding associations between sleep duration, insomnia symptoms, and sleep-disordered breathing across a range of health outcomes in postmenopausal women. *Sleep health* **6**, 48-59, doi:10.1016/j.sleh.2019.09.005 (2020).

B) The research factors were not sleep characteristics that we were interested in, such as insomnia, sleep disturbance, and obstructive sleep apnea (n=24)

7 Rada, R. Obstructive sleep apnea and head and neck neoplasms. *Otolaryngology-Head and Neck Surgery* **132**, 794-799, doi:10.1016/j.otohns.2004.12.002 (2005).

8 Li, Y. *et al.* The association between insomnia symptoms and mortality: A prospective study and meta-analysis. *Sleep* **36**, A195 (2013).

9 Li, Y. *et al.* Association between insomnia symptoms and mortality: a prospective study of U.S. men. *Circulation* **129**, 737-746, doi:10.1161/circulationaha.113.004500 (2014).

10 Shantha, G. P. S., Kumar, A. A., Cheskin, L. J. & Pancholy, S. B. Association Between Sleep Disordered Breathing, Obstructive Sleep Apnea and Incident Cancer: A Systematic Review and Meta-Analysis. *Circulation* **130** (2014).

11 Shantha, G. P. S., Kumar, A. A., Cheskin, L. J. & Pancholy, S. B. Association between sleep-disordered breathing, obstructive sleep apnea, and cancer incidence: a systematic review and meta-analysis. *Sleep Medicine* **16**, 1289-1294, doi:10.1016/j.sleep.2015.04.014 (2015).

12 Campos-Rodriguez, F. Sleep-disordered breathing and cancer incidence: An association for the next decade? *Sleep Medicine* **16**, 1287-1288, doi:10.1016/j.sleep.2015.05.010 (2015).

13 Zhang, X. B., Peng, L. H., Lyu, Z., Jiang, X. T. & Du, Y. P. Obstructive sleep apnoea and the incidence and mortality of cancer: a meta-analysis. *Eur J Cancer Care (Engl)* **26**,

doi:10.1111/ecc.12427 (2017).
14 Ge, L. *et al.* Insomnia and risk of mortality from all-cause, cardiovascular disease, and cancer: Systematic review and meta-analysis of prospective cohort studies. *Sleep Medicine Reviews* **48**, doi:10.1016/j.smrv.2019.101215 (2019).
15 Shi, T. *et al.* Does insomnia predict a high risk of cancer? A systematic review and meta-analysis of cohort studies. *J Sleep Res* **29**, e12876, doi:10.1111/jsr.12876 (2020).
16 Imani, M. M. *et al.* Serum and Plasma Tumor Necrosis Factor Alpha Levels in Individuals with Obstructive Sleep Apnea Syndrome: A Meta-Analysis and Meta-Regression. *Life-Basel* **10**, doi:10.3390/life10060087 (2020).
17 Sutherland, R. & Platt, J. THE ASSOCIATION BETWEEN OBSTRUCTIVE SLEEP APNEA AND CANCER INCIDENCE AND MORTALITY: A SYSTEMATIC REVIEW AND META-ANALYSIS. *Sleep* **43**, A229-A229 (2020).
18 Wei, L., Han, N., Sun, S., Ma, X. & Zhang, Y. Sleep-disordered breathing and risk of the breast cancer: A meta-analysis of cohort studies. *Int J Clin Pract* **75**, e14793, doi:10.1111/ijcp.14793 (2021).
19 Cheng, H. & Li, D. Investigation into the association between obstructive sleep apnea and incidence of all-type cancers: a systematic review and meta-analysis. *Sleep Med* **88**, 274-281, doi:10.1016/j.sleep.2021.05.031 (2021).
20 Cheng, L. J., Guo, H., Zhang, Z. L., Yao, Y. Y. & Yao, Q. L. Obstructive sleep apnea and incidence of malignant tumors: a meta-analysis. *Sleep Medicine* **84**, 195-204, doi:10.1016/j.sleep.2021.05.029 (2021).
21 Elfanagely, Y., Atsawarungruangkit, A., Scharfen, J., Pavlech, L. & Moss, S. F. Association Between Obstructive Sleep Apnea and Barrett's Esophagus: A Systematic Review and Meta-Analysis. *Dig Dis Sci* **66**, 3689-3697, doi:10.1007/s10620-020-06709-1 (2021).
22 Tan, N. K. W. *et al.* Obstructive sleep apnea and breast cancer incidence: A systematic review and meta-analysis. *Annals of Oncology* **32**, S89-S89, doi:10.1016/j.annonc.2021.03.172 (2021).
23 Tan, N. K. W. *et al.* The association of obstructive sleep apnea with melanoma incidence and mortality: a meta-analysis of 5,276,451 patients. *Sleep Med* **88**, 213-220, doi:10.1016/j.sleep.2021.10.027 (2021).
24 Varallo, G. *et al.* Sleep disturbances and sleep disorders as risk factors for chronic postsurgical pain: A systematic review and meta-analysis. *Sleep Med Rev* **63**, 101630, doi:10.1016/j.smrv.2022.101630 (2022).
25 Ma, H., Zhang, X., Han, J. & Li, F. Sleep-disordered breathing and risk of lung cancer: a meta-analysis longitudinal follow-up studies. *Eur J Cancer Prev* **31**, 245-252, doi:10.1097/cej.0000000000000707 (2022).
26 Chen, M. X. *et al.* Obstructive sleep apnea and the risk of mortality in patients with lung cancer: a meta-analysis. *Sleep and Breathing* **26**, 559-566, doi:10.1007/s11325-021-02416-x (2022).
27 Cheong, A. J. Y. *et al.* Obstructive Sleep Apnea and Lung Cancer A Systematic Review and Meta-Analysis. *Annals of the American Thoracic Society* **19**, 469-475, doi:10.1513/AnnalsATS.202108-960OC (2022).
28 Tan, B. K. J. *et al.* Association of obstructive sleep apnea with thyroid cancer incidence: a systematic review and meta-analysis. *Eur Arch Otorhinolaryngol*, doi:10.1007/s00405-

022-07457-w (2022).
29 Tan, B. K. J. *et al.* Association of obstructive sleep apnea and nocturnal hypoxemia with all-cancer incidence and mortality: a systematic review and meta-analysis. *Journal of Clinical Sleep Medicine* **18**, 1427-1440, doi:10.5664/jcsm.9772 (2022).
30 Yap, D. W. T. *et al.* The Association of Obstructive Sleep Apnea With Breast Cancer Incidence and Mortality: A Systematic Review and Meta-analysis. *J Breast Cancer* **25**, 149-163, doi:10.4048/jbc.2022.25.e11 (2022).

C) The research outcomes were not cancer-related events (n=3)

31 Youngstedt, S. D. & Kripke, D. F. Long sleep and mortality: Rationale for sleep restriction. *Sleep Medicine Reviews* **8**, 159-174, doi:10.1016/j.smrv.2003.10.002 (2004).
32 Shen, X., Wu, Y. & Zhang, D. Nighttime sleep duration, 24-hour sleep duration and risk of all-cause mortality among adults: a meta-analysis of prospective cohort studies. *Sci Rep* **6**, 21480, doi:10.1038/srep21480 (2016).
33 Liu, T. Z. *et al.* Sleep duration and risk of all-cause mortality: A flexible, non-linear, meta-regression of 40 prospective cohort studies. *Sleep Medicine Reviews* **32**, 28-36, doi:10.1016/j.smrv.2016.02.005 (2017).

D) Abstracts, letters or editorial comments (n=5)

34 Wang, X., Cairns, B. J. & Travis, R. C. SLEEP DURATION AND BREAST CANCER RISK: A META-ANALYSIS. *Journal of Epidemiology and Community Health* **64**, A32-A32, doi:10.1136/jech.2010.120956.80 (2010).
35 Iftikhar, I. H., Sri, V. C., Albisher, E. & Paul, G. Sleep duration and cancer risk: An updated meta-analysis of prospective studies. *Sleep* **39**, A268 (2016).
36 Wong, A. T. Y. *et al.* Sleep duration and breast cancer incidence: results from the million women study and a meta-analysis of published prospective studies. *Sleep Medicine* **64**, S421-S422, doi:10.1016/j.sleep.2019.11.1171 (2019).
37 Kawada, T. Total sleep time and all cancer mortality: a meta-analysis. *Sleep Medicine* **68**, 96, doi:10.1016/j.sleep.2019.12.029 (2020).
38 Ling, L., Shah, S., Hussain, J., Stranges, S. & Anderson, K. Night shift work, sleep quality and risk of endocrine-related cancer: a systematic review. *European Journal of Public Health* **30**, V956-V956 (2020).

E) Not the systematic review or meta-analysis with the largest data set (n=10)

39 Gallicchio, L. & Kalesan, B. Sleep duration and mortality: A systematic review and meta-analysis. *Journal of Sleep Research* **18**, 148-158, doi:10.1111/j.1365-2869.2008.00732.x (2009).
40 Lu, Y., Tian, N., Yin, J., Shi, Y. & Huang, Z. Association between sleep duration and cancer risk: a meta-analysis of prospective cohort studies. *PLoS One* **8**, e74723, doi:10.1371/journal.pone.0074723 (2013).
41 Zhao, H. *et al.* Sleep duration and cancer risk: A systematic review and meta-analysis of prospective studies. *Asian Pacific Journal of Cancer Prevention* **14**, 7509-7515, doi:10.7314/APJCP.2013.14.12.7509 (2013).
42 Qin, Y., Zhou, Y., Zhang, X., Wei, X. & He, J. Sleep duration and breast cancer risk: A

meta-analysis of observational studies. *International Journal of Cancer* **134**, 1166-1173, doi:10.1002/ijc.28452 (2014).

43 Yang, W. S., Deng, Q., Fan, W. Y., Wang, W. Y. & Wang, X. Light exposure at night, sleep duration, melatonin, and breast cancer: A dose-response analysis of observational studies. *European Journal of Cancer Prevention* **23**, 269-276, doi:10.1097/CEJ.0000000000000030 (2014).

44 He, C., Anand, S. T., Ebell, M. H., Vena, J. E. & Robb, S. W. Circadian disrupting exposures and breast cancer risk: a meta-analysis. *Int Arch Occup Environ Health* **88**, 533-547, doi:10.1007/s00420-014-0986-x (2015).

45 Ma, Q. Q., Yao, Q., Lin, L., Chen, G. C. & Yu, J. B. Sleep duration and total cancer mortality: a meta-analysis of prospective studies. *Sleep Medicine* **27-28**, 39-44, doi:10.1016/j.sleep.2016.06.036 (2016).

46 Lu, C. *et al.* Long-Term Sleep Duration as a Risk Factor for Breast Cancer: Evidence from a Systematic Review and Dose-Response Meta-Analysis. *Biomed Res Int* **2017**, 4845059, doi:10.1155/2017/4845059 (2017).

47 Li, Y. *et al.* Association between total sleep time and all cancer mortality: non-linear dose-response meta-analysis of cohort studies. *Sleep Med* **60**, 211-218, doi:10.1016/j.sleep.2019.03.026 (2019).

48 Wilunda, C. *et al.* Sleep duration and risk of cancer incidence and mortality: A pooled analysis of six population-based cohorts in Japan. *International Journal of Cancer*, doi:10.1002/ijc.34133 (2022).

Table S3. Basic characteristics of meta-analyses that assess sleep characteristics and cancer-related outcomes

Outcome	Source	Comparison	Explanation of exposure	Study design	No. of datasets	No. of participants	Summary effects (95% CI)		P_{Egger}	$P_{\text{heterogeneity}}$
							Random effects	Fixed effects		
**Sleep duration**										
All-cancer risk	Chen Y, 2018	short vs. ref	Ref: 5~8, 6~7, 6~8, 6.1~8.9, 6.6~7.4, 7, 7~7.5, 7~7.9, 7~8, 7~9, 8, and 8~9h (per night or 24 h). Short: 3~5, 3~6, < 5, ≤ 5, < 5.9, < 6, ≤ 6, ≤ 6.5, < 7, and ≤ 7h (per night or 24 h). Long: >7, > 8, ≥ 8, > 9, ≥ 9, >	CS and CCS	65	6609205	1.01 (0.97, 1.05)	0.99 (0.96, 1.02)	0.051	0.015
		long vs. ref		CS and CCS	65	6609205	1.02 (0.97, 1.07)	1.01 (0.97, 1.04)	0.935	0.010
Skin cancer risk	Chen Y, 2018	short vs. ref		CS	6	503211	0.93 (0.88, 1.00)	0.93 (0.88, 1.00)	0.974	0.481
		long vs. ref		CS	6	503211	0.92 (0.78, 1.10)	0.93 (0.83, 1.06)	0.854	0.287
Colorectal cancer risk	Chen Y, 2018	short vs. ref		CS	6	581111	1.05 (0.92, 1.19)	1.05 (0.95, 1.15)	0.542	0.151
		long vs. ref		CS	6	581111	1.21 (1.08, 1.34)	1.21 (1.08, 1.34)	0.170	0.555
Ovarian cancer risk	Chen Y, 2018	short vs. ref		CS	3	271215	1.05 (0.72, 1.53)	1.01 (0.82, 1.25)	0.727	0.166
		long vs. ref		CS	3	271215	0.84 (0.46, 1.52)	0.80 (0.56, 1.15)	0.686	0.079
Endometrial cancer risk	Chen Y, 2018	short vs. ref		CS	3	274192	0.98 (0.82, 1.17)	0.95 (0.84, 1.07)	0.079	0.135
		long vs. ref		CS	3	274192	1.06 (0.83, 1.34)	1.06 (0.83, 1.34)	0.917	0.589
Thyroid cancer risk	Chen Y, 2018	short vs. ref		CS	3	440118	1.11 (0.64, 1.93)	0.89 (0.71, 1.12)	0.262	0.058
		long vs. ref		CS	3	440118	0.95 (0.63, 1.45)	0.95 (0.63, 1.45)	0.025	0.539
Lung cancer risk	Chen Y, 2018	short vs. ref		CS	5	422406	1.04 (0.88, 1.22)	1.02 (0.91, 1.13)	0.240	0.115
		long vs. ref		CS	5	422406	1.01 (0.83, 1.23)	0.98 (0.86, 1.11)	0.455	0.144
Prostate cancer risk	Liu R, 2020	short vs. ref		CS	6	283430	0.99 (0.91, 1.07)	0.99 (0.91, 1.07)	0.466	0.530
		long vs. ref		CS	6	278932	0.88 (0.75, 1.04)	0.94 (0.87, 1.02)	0.241	0.044

Breast cancer risk	Wong ATY, 2021	short vs. ref	10, ≥ 10, > 10.2, and 10~12h (per night or 24 h).	CS	15	1476606	0.99 (0.97, 1.01)	0.99 (0.98, 1.01)	0.347	0.369
		long vs. ref		CS	15	1476606	1.00 (0.96, 1.04)	1.01 (0.98, 1.04)	0.065	0.328
All-cancer mortality	Stone CR, 2019	short vs. ref		CS	24	1128283	1.03 (1.00, 1.06)	1.03 (1.00, 1.06)	0.140	0.450
		long vs. ref		CS	26	1138118	1.09 (1.04, 1.13)	1.08 (1.04, 1.13)	0.045	0.385
Lung cancer mortality	Stone CR, 2019	short vs. ref		CS	16	374058	1.21 (1.10, 1.33)	1.22 (1.15, 1.29)	0.954	0.002
		long vs. ref		CS	16	358715	1.65 (1.36, 2.00)	1.55 (1.44, 1.66)	0.302	< 0.001
Breast cancer mortality	Stone CR, 2019	short vs. ref		CS	5	196578	1.08 (0.86, 1.36)	1.04 (0.91, 1.19)	0.496	0.053
		long vs. ref		CS	5	195464	1.11 (0.74, 1.67)	1.26 (1.02, 1.56)	0.304	0.026
Prostate cancer mortality	Stone CR, 2019	short vs. ref		CS	4	426956	1.02 (0.88, 1.18)	1.02 (0.88, 1.18)	0.770	0.769
		long vs. ref		CS	5	429529	0.94 (0.66, 1.32)	0.93 (0.75, 1.17)	0.980	0.133
**Sleep quality**										
All-cancer risk	Erren TC, 2016	poor vs. good	Judging through questionnaires on insomnia, sleep disorders, sleep problems, etc	CS and CCS	11	510593	1.12 (1.03, 1.21)	1.07 (1.03, 1.11)	0.050	0.014
Breast cancer risk	Erren TC, 2016	poor vs. good		CS and CCS	4	198472	1.03 (0.94, 1.13)	1.01 (0.96, 1.07)	0.204	0.258
**Napping**										
All-cancer risk	Erren TC, 2016	“yes” vs. “no”	“yes”: yes or often; “no”: no or rarely	CS and CCS	5	1665683	1.03 (0.95, 1.11)	1.06 (1.04, 1.08)	0.448	< 0.001
All-cancer mortality	Zhong G, 2015	“yes” vs. “no”		CS	4	92059	1.07 (0.99, 1.15)	1.06 (0.99, 1.12)	0.560	0.349

Abbreviation: CCS: case-control study; CI, confidence interval; CS: cohort study.

Table S4. Sensitivity analysis for evidence evaluation on sleep characteristics and cancer-related outcomes

**Outcome**	**Source**	**Comparison**	**No. of data sets**	**Quality evaluation of evidence**							
				***P* random-effects**	**No. of cases/outcomes**	***P* the largest study ***	**I^2 (95% CI)**	**95% prediction interval**	**Small-study effects**	**Excess significance bias**	**Evidence class**
**Sleep duration**											
All-cancer risk $	Chen Y, 2018	short vs. ref	61	> 0.05	> 1000	> 0.05	28.1 (1, 48)	0.86, 1.16	no	no	Non-significant
		long vs. ref	61	> 0.05	> 1000	> 0.05	28.3 (1, 48)	0.82, 1.23	no	no	Non-significant
All-cancer risk &	Zhao H, 2013	short vs. ref	10	> 0.05	> 1000	> 0.05	63.8 (28, 82)	0.69, 1.62	no	no	Non-significant
		long vs. ref	13	> 0.05	> 1000	> 0.05	67.6 (42, 82)	0.55, 1.52	yes	no	Non-significant
Prostate cancer risk &	Chen Y, 2018	short vs. ref	4	> 0.05	> 1000	> 0.05	0.0 (0, 85)	0.77, 1.17	no	no	Non-significant
		long vs. ref	4	> 0.05	> 1000	> 0.05	70.9 (17, 90)	0.19, 2.92	no	no	Non-significant
Breast cancer risk &	Chen Y, 2018	short vs. ref	12	> 0.05	> 1000	> 0.05	46.1 (0, 72)	0.83, 1.21	no	no	Non-significant
		long vs. ref	12	> 0.05	> 1000	> 0.05	51.0 (5, 75)	0.77, 1.34	no	no	Non-significant
Colorectal cancer risk &	Erren TC, 2016	short vs. ref	4	> 0.05	> 1000	> 0.05	0.0 (0, 85)	0.87, 1.27	no	no	Non-significant
		long vs. ref	4	$< 10^{-3}$ but $> 10^{-6}$	> 1000	< 0.05	0.0 (0, 85)	0.98, 1.46	no	no	Suggestive
All-cancer mortality &	Li Y, 2019	short vs. ref	20	> 0.05	> 1000	> 0.05	0.0 (0, 48)	0.99, 1.05	no	no	Non-significant
		long vs. ref	20	$< 10^{-3}$ but $> 10^{-6}$	> 1000	> 0.05	0.0 (0, 48)	1.02, 1.08	no	no	Suggestive
**Sleep quality**											

All-cancer risk $	Erren TC, 2016	poor vs. good	9	< 0.05 but > 10^{-3}	> 1000	> 0.05	51.0 (0, 77)	0.92, 1.33	no	no	Weak
Breast cancer risk $	Erren TC, 2016	poor vs. good	3	> 0.05	> 1000	> 0.05	50.3 (0, 86)	0.22, 5.02	no	no	Non-significant
**Napping**											
All-cancer risk $	Erren TC, 2016	yes vs. no	4	> 0.05	> 1000	> 0.05	87.2 (69, 95)	0.78, 1.42	no	no	Non-significant

Abbreviation: CI, confidence interval.

* The largest study: the study with the smallest standard error in a meta-analysis.

$ Sensitivity analyses were conducted after case-control studies were removed.

& Sensitivity analyses were conducted using eligible systematic reviews or meta-analyses with the second largest data set.

Outcome	Comparison	Total evidence class	Evidence class in subgroups				
			North America	Europe	Asia	24h sleep	Night sleep
**Sleep duration**							
All-cancer risk	short vs. ref	Non-significant	Non-significant	Non-significant	Weak	Non-significant	Non-significant
	long vs. ref	Non-significant	Non-significant	Non-significant	Non-significant	Non-significant	Non-significant
Skin cancer risk	short vs. ref	Weak	Weak	- *	-	Weak	-
	long vs. ref	Non-significant	Non-significant	-	-	Non-significant	-
Colorectal cancer risk	short vs. ref	Non-significant	Non-significant	-	-	Non-significant	Non-significant
	long vs. ref	Suggestive	Suggestive	-	-	Weak	Weak
Endometrial cancer risk	short vs. ref	Non-significant	Non-significant	-	-	-	-
	long vs. ref	Non-significant	Non-significant	-	-	-	-
Thyroid cancer risk	short vs. ref	Non-significant	Non-significant	-	-	-	Non-significant
	long vs. ref	Non-significant	Non-significant	-	-	-	Non-significant
Lung cancer risk	short vs. ref	Non-significant	Non-significant	-	-	-	Non-significant
	long vs. ref	Non-significant	Non-significant	-	-	-	Non-significant
Prostate cancer risk	short vs. ref	Non-significant	Non-significant	-	-	Non-significant	-
	long vs. ref	Non-significant	Non-significant	-	-	Non-significant	-
Breast cancer risk	short vs. ref	Non-significant	Non-significant	-	Non-significant	Non-significant	Non-significant
	long vs. ref	Non-significant	Non-significant	-	Non-significant	Non-significant	Non-significant
All-cancer mortality	short vs. ref	Non-significant	Weak	Non-significant	Non-significant	Non-significant	Non-significant
	long vs. ref	Suggestive	Non-significant	Non-significant	Suggestive	Weak	Weak
Lung cancer mortality	short vs. ref	Suggestive	-	-	Suggestive	Convincing	Non-significant
	long vs. ref	Suggestive	-	-	Suggestive	Highly suggestive	Non-significant
Breast cancer mortality	short vs. ref	Non-significant	Non-significant	-	-	-	Non-significant
	long vs. ref	Non-significant	Non-significant	-	-	-	Non-significant
Prostate cancer mortality	long vs. ref	Non-significant	-	-	-	Non-significant	-
**Sleep quality**							
All-cancer risk	poor vs. good	Weak	Non-significant	Weak	-	-	-
**Napping**							
All-cancer risk	"yes" vs. "no"	Non-significant	-	Non-significant	-	-	-

Upgrade

Downgrade

Unchanged

Null value

Figure S1. Change of evidence class of associations between sleep characteristics and cancer-related outcomes in subgroup analyses compared to main findings. * Null value due to < 3 datasets in subgroups.
